# Echinacea Reduces Antibiotics by Preventing Respiratory Infections: A Meta-Analysis (ERA-PRIMA)

**DOI:** 10.3390/antibiotics13040364

**Published:** 2024-04-16

**Authors:** Giuseppe Gancitano, Nicola Mucci, Rainer Stange, Mercedes Ogal, Selvarani Vimalanathan, Mahfuza Sreya, Anthony Booker, Bushra Hadj-Cherif, Werner C. Albrich, Karin Woelkart-Ardjomand, Samo Kreft, Wim Vanden Berghe, Godehard Hoexter, Andreas Schapowal, Sebastian L. Johnston

**Affiliations:** 1 1st Carabinieri Paratrooper Regiment “Tuscania”, Italian Ministry of Defence, 57127 Livorno, Italy; 2 Department of Experimental and Clinical Medicine, University of Florence, 50134 Florence, Italy; nicola.mucci@unifi.it; 3 Institute of Social Medicine, Epidemiology and Health Economics, Charité-Universitätsmedizin Berlin, Humboldt-Universität zu Berlin and Berlin Institute of Health, 10117 Berlin, Germany; rainer.stange@immanuelalbertinen.de; 4 Pediatric Clinic Brunnen, 6440 Brunnen, Switzerland; mercedes@ogal.ch; 5 Pathology & Laboratory Medicine, Faculty of Medicine, University of British Columbia, Vancouver, BC V6T 2B5, Canada; selvarani.vimalanathan@ubc.ca (S.V.); mahfuza.sreya@ubc.ca (M.S.); 6 Research Group for Optimal Health, School of Life Sciences, College of Liberal Arts and Sciences, University of Westminster, London W1B 2HW, UK; a.booker@westminster.ac.uk (A.B.); w1721396@my.westminster.ac.uk (B.H.-C.); 7 Research Group ‘Pharmacognosy and Phytotherapy’, UCL School of Pharmacy, London WC1N 1AX, UK; 8 Division of Infectious Disease, Infection Prevention and Travel Medicine, Cantonal Hospital, 9000 St. Gallen, Switzerland; werner.albrich@kssg.ch; 9 Department of Pharmacognosy, Institute of Pharmaceutical Sciences, University of Graz, 8010 Graz, Austria; ka.woelkart@uni-graz.at; 10 Faculty of Pharmacy, University of Ljubljana, 1000 Ljubliana, Slovenia; samo.kreft@ffa.uni-lj.si; 11 Department of Biomedical Sciences, University of Antwerp, 2000 Antwerp, Belgium; wim.vandenberghe@uantwerpen.be; 12 Statistical Consulting Godehard Hoexter, 79100 Freiburg, Germany; godehard.hoexter@gmail.com; 13 Allergy Clinic, CH-7302 Landquart, Switzerland; andreas@schapowal.ch; 14 National Heart and Lung Institute, Imperial College London, London W2 1PG, UK

**Keywords:** *Echinacea*, prevention, respiratory tract infections, antibiotics, recurrent RTIs, complications

## Abstract

Respiratory tract infections (RTIs) are the leading cause of antibiotic prescriptions, primarily due to the risk for secondary bacterial infections. In this study, we examined whether *Echinacea* could reduce the need for antibiotics by preventing RTIs and their complications, and subsequently investigated its safety profile. A comprehensive search of EMBASE, PubMed, Google Scholar, Cochrane DARE and clinicaltrials.gov identified 30 clinical trials (39 comparisons) studying *Echinacea* for the prevention or treatment of RTIs in 5652 subjects. *Echinacea* significantly reduced the monthly RTI occurrence, risk ratio (RR) 0.68 (95% CI 0.61–0.77) and number of patients with ≥1 RTI, RR = 0.75 [95% CI 0.69–0.81] corresponding to an odds ratio 0.53 [95% CI 0.42–0.67]. *Echinacea* reduced the risk of recurrent infections (RR = 0.60; 95% CI 0.46–0.80), RTI complications (RR = 0.44; 95% CI 0.36–0.54) and the need for antibiotic therapy (RR = 0.60; 95% CI 0.39–0.93), with total antibiotic therapy days reduced by 70% (IRR = 0.29; 95% CI 0.11–0.74). Alcoholic extracts from freshly harvested *Echinacea purpurea* were the strongest, with an 80% reduction of antibiotic treatment days, IRR 0.21 [95% CI 0.15–0.28]. An equal number of adverse events occurred with *Echinacea* and control treatment. *Echinacea* can safely prevent RTIs and associated complications, thereby decreasing the demand for antibiotics. Relevant differences exist between *Echinacea* preparations.

## 1. Introduction

Despite advances in pathological understanding, hygienic improvements and vaccination technology, respiratory tract infections (RTIs) are still the most frequent illnesses worldwide. They are divided into upper RTIs (URTIs), which affect the naso-pharynx and sinuses, and lower RTIs (LRTIs), which affect the trachea, bronchi and lungs [1]. A study performed by the Global Burden of Diseases, Injuries and Risk Factors (GBD) estimated that by 2019, 17.2 billion cases (or 42.8% of all worldwide diseases) were a consequence of URTIs, with a high prevalence in countries with high sociodemographic indices [2]. The same study attributed 291.7 million cases to LRTIs, of which approximately 1% were fatal [3]. In 2019, LRTIs were the leading infectious cause of death [4].

Approximately one-third of all RTIs affect children below five years of age, of which a disproportionally high number of 0.7 million cases are lethal. A higher fatality rate is also reported for elderly people and immunocompromised patients [5]. These numbers do not account for the recent COVID-19 pandemic that caused an estimated 677 million infections and 6.9 million deaths worldwide [6]. 

Containment measures like social distancing and hygiene not only curbed overall viral infections but also secondary bacterial respiratory infections and, importantly, the worldwide use of antibiotics—indicating a close correlation between those factors [7]. Suspension of those containment measures brought antibiotic use back to pre-pandemic levels and, although COVID-19 is understood as a viral illness that is rarely associated with bacteria (10%), up to 75% of infections were treated with antibiotics [8,9]. 

The prevention of RTIs may be achieved by taking *Echinacea* species, as antiviral and immune-modulatory actions have been reported [10,11]. Great heterogeneity exists between different preparations, but for alcoholic extracts, recent literature found a wide spectrum of activity against enveloped respiratory viruses, including influenza viruses, respiratory syncytial virus (RSV), coronaviruses and SARS-CoV-2 [10]. Activation of interferon signaling, chemotaxis and anti-inflammatory actions constitute the immune supportive effects of the medicinal plant [11,12]. Clinical benefits manifest not only in a reduced risk of RTIs but also of RTI relapses and secondary complications [13].

For the first time, a recent study in children demonstrated a benefit on the frequency of antibiotic prescriptions, showing a reduction by 76.3%, which was significant as a secondary outcome [14]. The aim of the current systematic review and meta-analysis was to test the hypothesis that taking *Echinacea* could reduce not only recurrent RTI episodes but also RTI complications and, further, that this reduction would lead to a reduced need for antibiotic prescriptions. In addition, we investigated the safety profile by studying the occurrence of adverse events (AEs) upon *Echinacea* therapy.

## 2. Results

**Figure 1:**
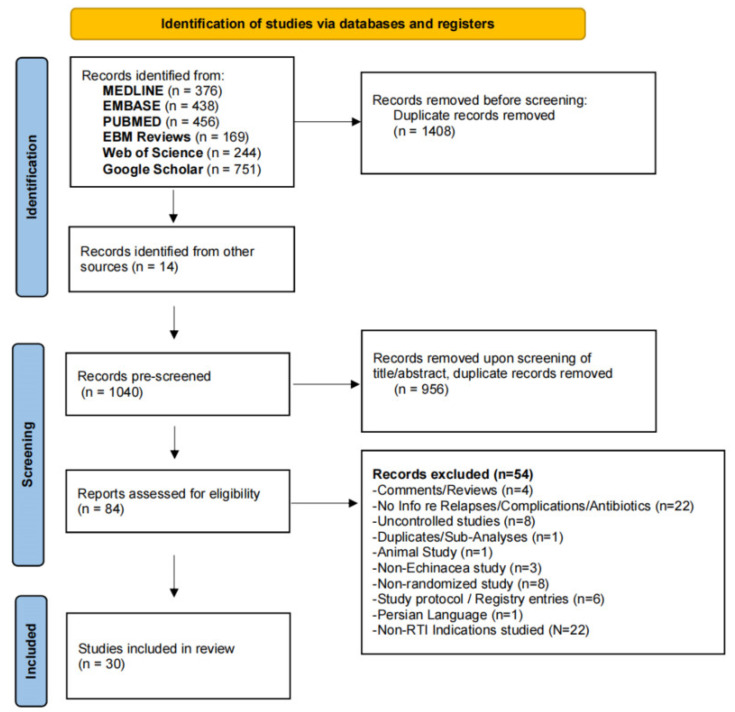
Flow chart of included and excluded studies.

Our systematic literature search yielded a total of 2434 hits from screened databases, whereas another 14 were identified from reviewing reference lists of review articles and study registers (Figure 1). 

After removing duplicates (*n* = 1408), records were selected based on title/abstract interpretation, leaving 84 articles overall, of which *n* = 54 did not describe original work, contained no information regarding RTIs (complications) or usage of antibiotics or were not controlled. 

### 2.1. Study Characteristics

Overall, a total of 30 clinical studies were included in our analysis, reporting on 39 comparisons of *Echinacea* preparations with a control group. In 22 trials, *Echinacea* was investigated for prevention of RTIs (27 comparisons [14,15,16,17,18,19,20,21,22,23,24,25,26,27,28,29,30,31,32,33,34,35]) while in 8 trials, *Echinacea* was studied for the acute treatment (12 comparisons [36,37,38,39,40,41,42,43]). Taylor et al. (2003) [42] and Sumer et al. (2023) [37] allowed for a repetitive therapy of up to three episodes over a prolonged observational time [37,42]. Six prevention studies administered *Echinacea* for a shorter period of equal or less than one month [21,24,25], three of which employed an artificial inoculation method [16,31,33], whereas the remaining studies employed longer treatment periods between six weeks to five months [14,15,17,18,19,20,22,23,26,27,28,29,30,32,34,42]. Awad et al. applied an interval preventive therapy of 6 × 10 days throughout half a year [29]. Weber et al. [35] presented a sub-analysis of the work by Taylor et al. [42], giving information on recurrent infections under *Echinacea* or placebo therapy.

A total of 21 studies investigated an *Echinacea* mono-product, with nine containing further ingredients like vitamin C, *Sambucus nigra*, *Nigella sativa*, *Thuja occidentalis, Baptisia tinctoria, propolis* or homeopathic dilutions as additives [17,18,19,20,22,25,30,39,43]. The majority of the 39 comparisons involved lipophilic *Echinacea purpurea* extracts based on alcoholic extractions, glycerol or hypercritical CO_2_ extractions [14,15,17,18,19,22,25,26,27,30,32,36,37,38,39]. Seven preparations contained *Echinacea purpurea* pressed-juices (hydrophilic) [21,23,24,31,33,40,42], whereas four preparations contained dried, powdered or unspecified *Echinacea* [16,28,29,43]. As anticipated, a great variety of *Echinacea* preparations were included in this analysis with the aim to investigate overarching evidence of activity for the medicinal plant. 

RTI was the studied indication, mostly detected as a patient-reported, physician/nurse-confirmed outcome [14,15,16,18,19,20,23,26,27,28,30,31,34,36,37,38,39,41,42,43]. This entity comprised the common cold, rhinitis, non-specified respiratory infections, flu-like infections or flu. More recent clinical studies also involved RT-PCR based confirmation of respiratory viruses [26,27,37,39] and three trials artificially induced infections through rhinovirus inoculation [16,31,33]. Seven studies included children below twelve years of age [14,18,22,25,29,41,42], whereas three trials researched *Echinacea* in children as young as one or two years [18,25,42]. 

With respect to safety, AEs were reported either as numbers of patients experiencing AEs or total number of AEs by 17 clinical studies [14,15,18,21,23,26,27,28,30,31,32,34,36,38,40,41,42]. Ogal et al. [14] reported a total of 105 AEs for 103 study subjects in the control group and we decided for this particular study to define the total number of AEs (105) rather than the sample size (103) as the denominator for assessment of the risk ratio (see Appendix A Table A3).

### 2.2. Risk of Bias

We employed the risk of bias tool by Cochrane (RoB2) to estimate the quality of included studies based on seven aspects addressing selection, performance and reporting biases. Our assessment of study quality was in principal agreement with results by David et al. [44], whereas additional literature was rated independently [45].

Some research was carried out before the implementation of the Consolidated Standards of Reporting Trials (CONSORT statement) in 1992 when reporting principles were still not elaborate yet. Where randomization was mentioned, details regarding sequence generation was sometimes missing [16,20,21,25,30,36]. In double-blind studies with low numbers of dropouts and principally healthy participants, we assumed a low risk for allocation concealment and performance bias (blinding of patients/personnel, attrition bias and incomplete outcomes). For open studies lacking placebo or using active control, a high risk for bias was principally suspected [17,19,20,22,25,29,41], unless blinding effectiveness was explicitly confirmed [15,27,37] and if an objective parameter was investigated (i.e., routine virus analytics from nasopharyngeal samples) [27]. Hence, high risk of bias was detected in at least one RoB2 domain in eleven studies, which consequently obtained inadequate quality ratings of <4 also according to Jadad [46] (see Appendix A Table A2) [17,19,20,22,25,27,29,34,36,40,41]. Those studies were dealt with separately in a sensitivity analysis. 

Selected studies mostly included healthy subjects, thus, the risk for imbalanced allocation and selection bias was expected to be low, as evidenced by demographic data given for most trials. Despite randomization, Wahl et al. obtained significantly heterogeneous groups for comparison [47] The article by Rahmati et al. provided an abstract in English but the main article was written in Arabic and was therefore excluded [48].

### 2.3. Results from Individual Studies

Results from individual studies are summarized in Appendix A Table A3. Information regarding RTI incidence was available in form of patients experiencing ≥ 1 episode/infection and/or the number of episodes/infections occurring throughout the observation period [14,15,16,17,18,19,21,22,23,24,25,26,27,28,29,30,31,32,33,34]. Since intervention durations varied greatly between studies (10 days–6 months), we normalized the latter parameter to monthly occurrence of RTI as well. Data pertaining to patients with recurrent infections/relapses or the number of recurrences/relapses was available from [14,15,18,23,25,26,30,42]. Those included classical prevention trials and acute therapy studies with appropriate follow-up periods. Finally, information on antibiotic use was gathered from 11 studies, either as number of patients treated with antibiotics, overall antibiotic treatment days or mean differences in antibiotic treatment days [14,18,25,26,27,36,37,39,41,42,43]. For all analyses, we conservatively commented on random rather than common/fixed effect model, while supplementing results for risk ratios (RR) by the odds ratio (OR), where appropriate.

### 2.4. Results of Meta-Analysis

#### 2.4.1. Prevention of Respiratory Tract Infections (RTIs)

**Figure 2:**
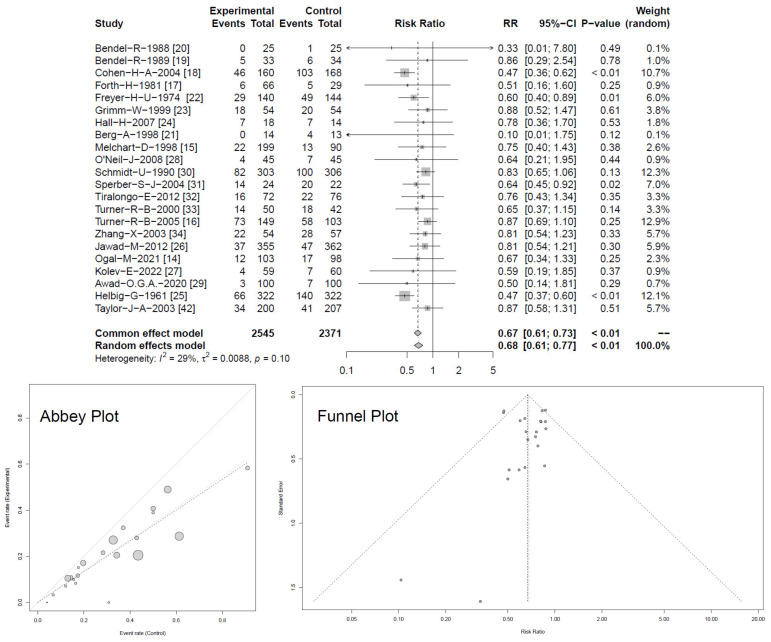
Forest plot showing meta-analysis of overall risk for occurrence of RTIs between groups with Abbey and Funnel plots, indicating low risk of publication bias (for clearer Abbey and Funnel plots see Appendix B Figure A2 and Figure A3). Shown are “events” (RTIs), “total” (participants) for *Echinacea* (“experimental”) and control, risk ratios (RR) employing a common and random effect model, heterogeneity (I^2^), confidence intervals (95%-CI), *p*-value and individual weight of respective studies.

As a first objective, the prevention of RTIs through *Echinacea* use was tested. Figure 2 shows the risk of RTIs normalized per treatment month and patient for *Echinacea* and control, referring to 4916 study subjects included in 22 studies [14,15,16,17,18,19,20,21,22,23,24,25,26,27,28,29,30,31,32,33,34,42]. All included studies point towards the superiority of *Echinacea* over control treatment. Effect sizes ranged between RR 0.10 and 0.88, where four studies reached a *p* < 0.05. Pooled effect sizes of individual studies (random effect model) yielded a significant risk ratio of RR = 0.68 [95% CI 0.61–0.77; *p* < 0.01], while a heterogeneity of I^2^ = 29% was considered to be low (τ^2^ = 0.0088; *p* = 0.1). 

**Figure 3:**
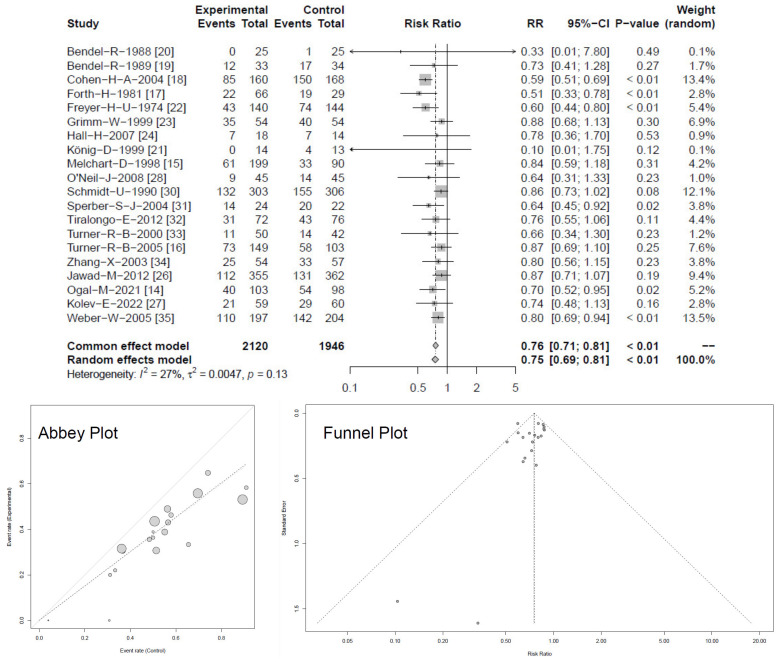
Forest plot showing meta-analysis of proportion of *Echinacea*-treated subjects with ≥1 RTI compared with control (for clearer Abbey and Funnel plots see Appendix B Figure A4 and Figure A5). Shown are “events” (pts with RTIs), “total” (participants) for *Echinacea* (“experimental”) and control, risk ratios (RR) employing a common and random effect model, heterogeneity (I^2^), confidence intervals (95%-CI), *p*-value and individual weight of respective studies.

Twenty studies reported numbers of participants experiencing one or more RTIs. When data were pooled in meta-analyses, heterogeneity decreased to I^2^ = 27% (τ^2^ = 0.0047, *p* = 0.13) and RR yielded 0.75 [95% CI 0.69–0.81; *p* < 0.01], respectively OR = 0.53 [95% CI 0.42–0.67; *p* < 0.01], see Figure 3 [14,15,16,17,18,19,20,21,22,23,24,26,27,28,30,35].

Again, all studies indicated superiority for *Echinacea*, of which six trials reported significant benefits, with *p* < 0.05. For both analyses (Figure 2 and Figure 3), random and common effect models provided similar and consistent results. Both Abbey and Funnel plots described a rather natural scatter of large and smaller studies showing a typical variation (confidence interval and standard deviation) experienced in such trials. Selection bias due to unpublished or possibly negative studies is not indicated. The dispersion of standard errors against estimated effect size also indicates the absence of asymmetry for the parameter monthly risk for RTIs. A similar picture was observed for number of participants with RTIs.

The above analysis contained several studies with high risk of bias in at least one section of the Cochrane RoB analysis, thus scoring less than four points in Jadad’s assessment. Exclusion of these potentially high-risk studies provided a result based on more reliable evidence without changing the estimated effect with RR = 0.75 [95% CI 0.64–0.87; *p* < 0.01] for RTI’s. Unexpectedly, the heterogeneity increased to significance with I^2^ = 40% (τ^2^ = 0.0052, *p* = 0.08), indicating that excluded studies, though lower in quality, stabilized the overall certainty of reported effect size estimates (see Appendix A, Table A4).

**Figure 4:**
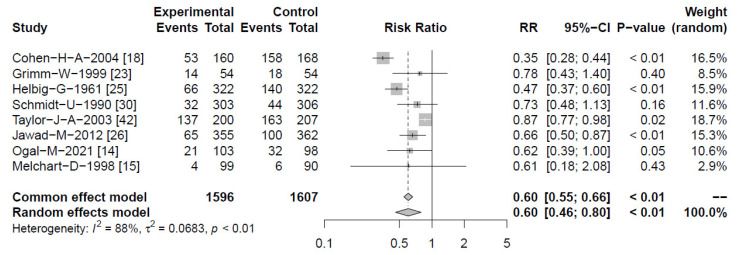
Forest plot showing meta-analysis of proportion of *Echinacea-*treated subjects experiencing recurrent RTIs/relapses compared with control. Shown are “events” (pts with recurrences), “total” (participants) for *Echinacea* (“experimental”) and control, risk ratios (RR) employing a common and random effect model, heterogeneity (I^2^), confidence intervals (95%-CI), *p*-value and individual weight of respective studies.

The risk for overall RTIs was lower than the risk for at least one episode. This was assumed to be a result of a diminished risk for recurrent infections and relapses, and was further explored. The risk for participants experiencing recurrent RTIs was calculated by pooling results from eight clinical studies comprising 3203 subjects, comparing *Echinacea* with control (mostly within a preventive scenario) [14,15,18,23,25,26,30,42]. A significant reduction of recurrences and relapses was found in the *Echinacea* group indicating a RR = 0.60 [95% CI 0.46–0.80; *p* < 0.01], but at significant heterogeneity of I^2^ = 88% (Figure 4).

#### 2.4.2. Reduction of RTI Complications

**Figure 5:**
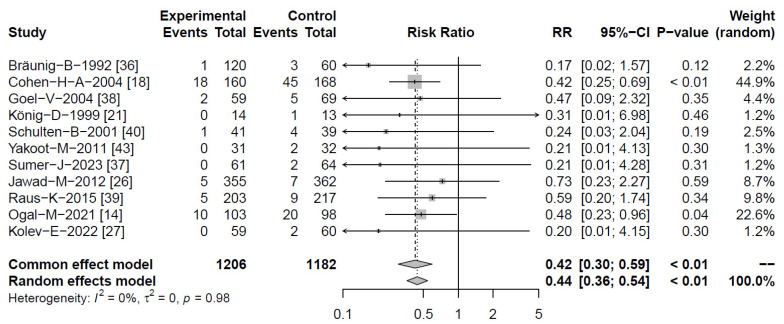
Forest plots showing meta-analysis of proportion of *Echinacea*-treated subjects experiencing complications compared with control. Shown are “events” (complications), “total” (participants) for *Echinacea* (“experimental”) and placebo risk ratios (RR) employing a common and random effect model, heterogeneity (I^2^), confidence intervals (95%-CI), *p*-value and individual weight of respective studies.

We pooled results pertaining to numbers of participants experiencing RTI complications, as well as the total numbers of complications occurring. Results were available from 11 [14,18,21,26,27,36,37,38,39,40,43] and 13 clinical studies [14,18,21,23,26,27,29,36,37,38,39,40,43], including 2388 and 2695 subjects, respectively. For both analyses, heterogeneity was either absent or moderate, pointing to a highly robust estimated effect size. A pronounced reduction of risk of complications was observed (RR = 0.44 [95% CI 0.36–0.54; *p* < 0.01]) which was highly comparable with the overall number of complications occurring. The two largest studies (both of good methodological quality with Jadad scores ≥ 4) provided results that were consistent with the estimated effect size upon meta-analysis. Consequently, the results from a sub-analysis including only high-quality studies provided a highly similar RR = 0.47 [95% CI 0.37–0.58] for participants with complications, again at the absence of heterogeneity. The estimated effect size was thus considered to be reliable (see Figure 5 and Appendix A Table A4). 

#### 2.4.3. Antibiotic Prescriptions

**Figure 6:**
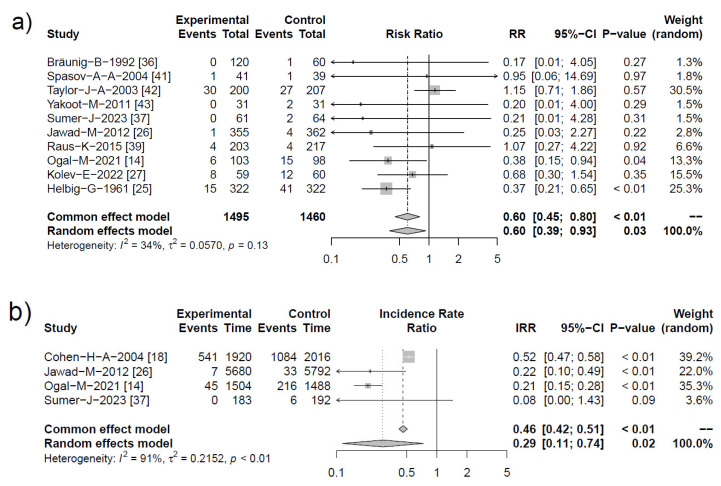
Forest plots showing meta-analysis of: (**a**) number of *Echinacea*-treated subjects receiving antibiotic therapy compared with control. Shown are “events” (pts or days with antibiotics), “total” (participants) for *Echinacea* (“experimental”) and placebo, risk ratios (RR) employing a common and random effect model, heterogeneity (I^2^), confidence intervals (95%-CI), *p*-value and individual weight of respective studies. (**b**) Number of overall antibiotic treatment days, showing individual risk ratio (IRR). Most studies reported the number of patients receiving antibiotic therapy.

Finally, we tested whether the use of *Echinacea* would also affect the need for antibiotics, as assessed by the number of participants treated with antibiotics, number of antibiotic treatment days and pooled mean differences between reported antibiotic treatment durations per individual. See Figure 6a,b for more information.

Results referring to antibiotic use were retrieved from ten clinical studies, of which seven trials were assigned a high methodological quality (Jadad score 5) [14,25,26,27,37,42,43] and three trials a poor methodological quality (Jadad score 1 and 2) [36,39,41]. Heterogeneity was calculated to be low with I^2^ = 34% and insignificant (*p* > 0.1). For the number of participants treated with antibiotics, both common and random effect models provided similar risks that were significant, with RR = 0.60 [95% CI 0.39–0.93; *p* = 0.03] for the random effects model (Figure 6a). Both Helbig and Taylor et al. [25,42] provided considerable cumulative weight of more than 50%, with the former study being of low quality and the latter describing a treatment study. Interestingly, upon exclusion of therapeutic studies [36,37,41,42,43], pure prevention studies provided an even more pronounced effect, with RR = 0.46 [95% CI 0.27–0.76; *p* = 0.01]. 

Figure 6b illustrates effects of *Echinacea* treatment on the overall duration of antibiotic therapy that was significantly reduced showing an individual risk ratio (IRR) = 0.29 [95% CI 0.11–0.74; *p* < 0.02]. Maximal benefits were achieved using a combined therapeutic approach with basic prevention dosing and dose-increase during acute illness, as shown by Cohen et al. [18]. The latter reported 541 versus 1084 days with antibiotic therapy for *Echinacea* and placebo, respectively, IRR 0.52 [95% CI 0.47–0.58; *p* < 0.01]. This result was only surpassed by Ogal et al. who showed an impressive 80% reduction from 216 to 45 antibiotic treatment days, IRR 0.21 [95% CI 0.15–0.28; *p* < 0.01] (Figure 6b) [14]. 

#### 2.4.4. Subanalyses

**Figure A1:**
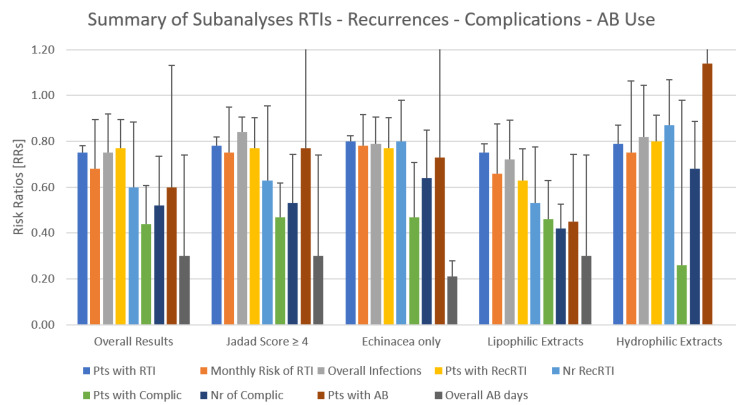
Graphical illustration of sub-analysis results.

As per registration of this meta-analysis, it was the clear intention to include all randomized controlled clinical trials investigating any *Echinacea* species, regardless of study design, quality, manufacturing method or addition of further supplements to the *Echinacea* product. It was also declared necessary to conduct sub-analysis on more discrete study selection criteria, yielding results which are discussed in the following section and provided in Appendix A Table A4 and Figure A1. Pooling of high-quality studies overall did not increase consistency (I^2^) but the magnitude of treatment effect and its statistical significance remained consistent with the overall meta-analyses throughout. The monthly risk for RTIs marginally increased to RR = 0.75 (*p* < 0.01), the effects on recurrent RTI, complications and most importantly, the 70% reduction of antibiotic treatment days remained stable and significant (Appendix A, Figure A1).

The separation of lipophilic from hydrophilic extracts revealed a clear distinction not only in terms of the monthly risk for RTIs (lipophilic vs hydrophilic RR = 0.66 [*p* < 0.01] vs. 0.75 [n.s.]), but also for recurrent RTIs (RR = 0.53 [*p* < 0.01] vs. 0.87 [n.s.]) and complications (RR = 0.42 [*p* < 0.01] vs. 0.68 [n.s.]), highlighting important differences between *Echinacea* preparations. 

There remains the question as to whether the addition of supplements would further enhance the benefits of *Echinacea.* As shown in Appendix A Table A4, results are inconsistent, where a trend towards higher monthly RTI RR values was balanced by an opposite trend for antibiotic use with a lower RR value and tighter CIs for *Echinacea*-only preparations. However, it is important to note that this analysis may be fundamentally influenced by the wide variety of *Echinacea* formulations, introducing more variance than additives, as shown previously.

#### 2.4.5. Adverse Events

**Figure 7:**
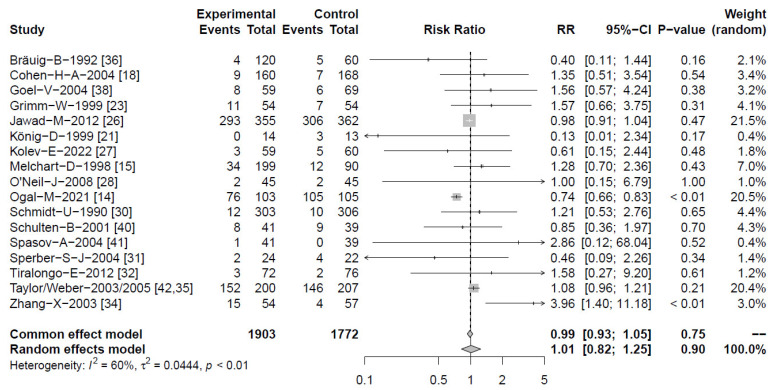
Information regarding occurrence of AEs from 17 clinical studies. Forest plots showing meta-analysis of proportion of *Echinacea*-treated subjects experiencing AEs compared with control. Shown are “events” (AEs), “total” (participants) for *Echinacea* (“experimental”) and placebo risk ratios (RR) employing a common and random effect model, heterogeneity (I^2^), confidence intervals (95%-CI), *p*-value and individual weight of respective studies.

Information regarding the occurrence of AEs was retrieved from 17 clinical studies (Figure 7) [14,15,18,21,23,26,27,28,30,31,32,34,36,38,40,41,42]. For both *Echinacea* and control, an overall number of 633 events were reported from 1903 and 1772 participants, respectively. The resulting risk and odds ratio for *Echinacea* versus control yielded highly similar values of OR = 0.99 [95% CI 0.64–1.47] and RR = 1.01 [0.85–1.20]; *p* = 0.90. The largest study by Jawad et al. [26] assessed the occurrence of AEs over four months long-term use, with similar figures for *Echinacea* and placebo [RR = 0.98 [95% CI 0.91–1.04]]. Ogal et al. investigated the same *Echinacea* preparation (Echinaforce^®^) to find significantly lower AEs in comparison with control (3 × 50 mg vitamin C), due to reduced RTI complications including otitis media or bronchitis [14]. Overall, AEs most often concerned mild gastro-intestinal complaints, which were self-limiting without medicinal intervention. 

## 3. Discussion

Global antibiotic use continues to rise despite governmental education programs (i.e., antibiotic stewardship) promoting their judicious use [49]. Every day of oral beta-lactam administration is estimated to increase the risk of carrying penicillin resistant pneumococci by 4% [50]. RTIs represent the most common reason for antibiotic use in not only the ambulatory but also inpatient and self-medication settings [51]. Reducing the antibiotic use for RTIs thus represents a unique opportunity to control the overuse of antibiotics in the future.

We investigated the potential of *Echinacea* species to prevent initial viral RTIs, thereby reducing secondary (likely bacterial) RTI complications and the need for antibiotics. Positive associations between *Echinacea* and the three levels of prevention could be demonstrated, showing a reduction of overall RTIs by ~32% at a RR = 0.68 [95% CI 0.61–0.77], of recurrences by approximately 40% at RR = 0.60 [95% CI 0.46–0.80] and of complications by approximately 56% at RR = 0.44 [95% CI 0.36–0.54]. These reductions resulted in approximately 40% fewer participants requiring antibiotics (RR = 0.60 [95% CI 0.39–0.93]) and a 70% reduction of antibiotic treatment days (RR = 0.30 [0.12–0.73]), both results on antibiotic use being statistically significant (*p* < 0.05). The former result included two trials [39,42] on the acute use of *Echinacea* and their exclusion aligned to figures on overall antibiotic treatment days. This supports the beneficial effects of long-term, preventative *Echinacea* supplementation. The difference between the two outcomes (antibiotic prescriptions vs. treatment duration) might also originate from using heterogeneous *Echinacea* preparations. Upon exclusion of hydrophilic extracts (pressed juices) as used in Taylor and Spasov [39,41], the RR = 0.45 [95% CI 0.30–0.66] for patients requiring antibiotics corresponded well with the value for antibiotic treatment days. 

Heterogeneity in meta-analysis is a crucial, yet common factor increasing variance to the estimated effect: varying manufacturing techniques (lipophilic versus hydrophilic extracts or further supplements), study designs (prevention versus acute treatment) or the methodological quality of included studies. We addressed this potential weakness by applying distinct selection criteria in function of the respective research question to attribute benefits to the various characteristics of heterogeneity in a sub-analysis. The differentiation between preparations used in trials more clearly revealed the correlation between RTIs, secondary complication and antibiotic use that was most convincingly demonstrated for lipophilic extracts. Those consisted mostly of alcoholic extracts from freshly harvested *Echinacea purpurea* herbs and roots (Echinaforce extract). This finding is consistent with data from Schapowal (2015) or Karsch–Voelk (2014), who also revealed important differences between *Echinacea* preparations [13,52]. An interesting observation was the fact that the two largest studies providing strongest effect sizes both investigated children preventively treated for three–four months using lipophilic preparations [14,18]. The RTI risks were very low with RRs = 0.47 and 0.67, recurrence risk RRs = 0.35 and 0.62, complication risk RRs = 0.42 and 0.48, leading to overall antibiotic treatment day IRR = 0.52 and 0.21 and fewer patients requiring antibiotics for the latter study, RR = 0.38 [0.15–0.94] (all *p*-values < 0.05). 

It is reasonable to assume that reported broad-spectrum antiviral effects of alcoholic fresh-plant *Echinacea* extracts contribute to the preventative benefit of such products [10]. This alone however may not fully explain the observed strong decrease on the level of antibiotic prescriptions. Immuno-modulation or tertiary antibacterial effects may support the recovery process of acute illness rendering antibiotic use unnecessary, however more research is warranted to further elucidate the accumulating trend from RTI prevention to antibiotic reduction [11,12].

Our results compare to previous meta-analyses from David and Cunningham (2019) [44], Karsch–Voelk et al. (2014) [52] and Shah et al. (2006) [53], each drawing conclusion on nine or ten prevention studies including less than 2000 participants [45,52,53]. Ten years ago, Karsch–Voelk et al. conferred, despite significant heterogeneity, a pooled RR for RTIs prevention of 0.83 [95% CI 0.75–0.92]. In a more recent analysis, David and Cunningham found a RR for RTI prevention by *Echinacea* of 0.78 [95% CI 0.68–0.88], whereas Shah expressed preventive effects in a pooled odds ratio of OR = 0.42 for the same parameter [95% CI 0.25–0.71]. The above analyses did not cover more recent literature or studies written in the German language, which our study did include.

Our results are based on data from 5652 study subjects included in 30 studies, yielding a comparable overall RR for RTIs of 0.68 [0.61–0.77, *p* < 0.01]. Similar to Shah, odds ratios found in our study were notably lower than risk ratios and, in our study, OR = 0.53 [0.42–0.67, *p* < 0.01] approached the value found by Shah, i.e., a reduction by over 50% in the absence of heterogeneity. 

This work demonstrates for the first time how *Echinacea* reduces antibiotic prescriptions and overall therapy duration on the level of a meta-analysis referring to randomized controlled clinical studies. Along with results on RTI incidence and duration, no previous meta-analyses investigated the sequence of incident RTI, RTI recurrences, RTI complications and the use of antibiotics, therefore no comparative effects are available. The strategy to reduce antibiotic use through RTI prevention is very promising and has been described similarly for influenza and pneumococcal vaccines, which are associated with a 10–40% reduction of antibiotic prescriptions or antibiotic days [54]. This effect has now been demonstrated to be applicable to *Echinacea* as well, while results shown for alcoholic fresh-plant *Echinacea* extracts (55–70%, Appendix A, Table A4) seem to exceed the effectiveness of the aforementioned vaccinations. A combined approach of vaccination plus *Echinacea* supplementation may provide even superior effects, however this would require confirmation in a confirmatory clinical study.

The safety profile of *Echinacea* was evaluated by previous meta-analyses along with the present research. David deduced a relative risk of RR = 1.09 [0.95–1.25] for participants reporting at least one adverse event [44] and Karsch–Voelk found a 2.4% dropout rate due to adverse events with *Echinacea* compared to 0.8% with placebo [52]. The latter, however, wrongly referenced the data from the largest trial by Jawad, therefore the result has to be questioned. We looked at overall occurring adverse events as safety parameter to find the very same number of adverse events occurring with *Echinacea* therapy and control, i.e., 633 AEs in a sample of 1903 and 1772 participants, OR = 0.99 [95% CI 0.636–1.47] and RR = 1.01 [0.82–1.25]; *p* = 0.90. These figures indicate a highly positive safety profile. In comparison with David and Karsch–Voelk, we looked at the total occurring adverse events rather than patients experiencing events [45,52]. However, both analyses underscore the very good safety profile of *Echinacea* extracts used for prevention and acute therapy. Taylor found an increase in allergic reactions for a pressed-juice formulation used in children, however this was not confirmed by Ogal or Cohen for lipophilic extracts, even upon long-term use over three–four months [14,18,42]. In most cases, adverse events were mild, self-limiting, gastrointestinal in nature and did not require medical intervention. 

Our analysis has limitations. First, we did not restrict publication date and also regarded early scientific studies prior to 2000, when reporting guidelines were not as strict. Hence, some publications received lower quality ratings. This however does not necessary indicate a low quality of the study per se. They may still provide valid results, a conclusion supported by the fact that analysis of only high-quality studies did not significantly change the overall result or decrease heterogeneity overall. 

Secondly, we carried out a series of sub-analysis accounting for the variability of included studies mentioned above. More extensive diversification would have been interesting but would have exceeded the scope of this work. Previous research focused on RTI prevention in immunologically susceptible individuals, finding better results in comparison to more robust subjects [13]. We did not explicitly investigate this population in more detail.

In conclusion, *Echinacea* could provide an effective and safe means to prevent RTIs and secondary complications to thereby significantly reduce the need for antibiotic prescriptions. However, due caution is implicated in the selection of the particular *Echinacea* product as differences may exist.

## 4. Methods

The purpose of this systematic review and meta-analysis was to investigate the potential of *Echinacea* species to prevent and treat RTI under the conditions of a RCT (randomized controlled trial). As an outcome, a trial had to compare at least one of the following between groups over the study period: RTIs, recurrent RTIs, complications of RTIs or use of antibiotics. Further, we collected reports on AEs for the assessment of *Echinacea*’s safety profile.

The protocol for this systematic review was registered on INPLASY under protocol ID: 4969-1. We carried out a comprehensive search of literature on EMBASE, PubMed, Google Scholar and Cochrane DARE from the respective databases’ day of inception until 30 June 2023 without restriction for language, publication status or particular patient groups and according to guidance [55,56]. An example literature search strategy is given in Table 1.

In addition, we screened the clinical trials register clinicaltrials.gov for completed studies with results on *Echinacea*. Some articles were available in German and thus literature was sought by any language and via screening bibliographies of identified trials and review articles. We did not include articles in Arabic [48]. Identified hits from the above searches were checked for duplicates using EndNote. Resulting hits were then displayed with abstract and title. Two review authors (GG, MS) were involved in the final selection of clinical articles studying *Echinacea* for treatment or prevention of RTIs in humans using a controlled setting. Random allocation to verum and control group was a prerequisite for inclusion in order to yield homogenous and comparable collectives. Articles were further regarded if information on (recurrent) RTIs, their complications and/or usage of antibiotics were reported. Two authors independently carried out the study selection process (GG, MS), whereas native speaking authors reviewed the German literature (GH, RS).

The resulting list of referenced literature was checked for consistency and completeness, and discrepancies were solved mutually. Study details were retrieved and data were extracted using a standard extraction form capturing authors, reference, study registration number, *Echinacea* species and manufacturing method, dosage, details on comparator, studied indication, methodology, patient sample, RTI occurrence, complications, antibiotic use and adverse events. We contacted investigators and sponsors of registered clinical studies in case of missing data. Results on recurring RTIs were deduced from the number of relapses/recurrences from the first dose of *Echinacea* until the end of treatment phase including any follow-up period, as defined by Schapowal (2015) [13]. Patients with and incidences of complications and/or bacterial superinfections were deduced from the same observation period retrieving reports for tonsillitis/pharyngitis, tracheitis, lymphadenitis, bronchitis, pneumonia, sinusitis, conjunctivitis, otitis media (acuta) or adverse events on respiratory system disorders. Regarding the use of antibiotics, we searched for the number of patients requiring antibiotics as well as treatment duration where available. 

According to pre-published protocol, our primary parameters were the odds for (recurrent) RTIs, of complications, respectively, the need for antibiotics during the time of *Echinacea* intervention and follow-up period in comparison with the control. Additionally, we evaluated results on the patient level, i.e., the number of patients reporting ≥1 RTI, recurrent RTIs, complications or those with antibiotic therapy. Accounting for the varying therapy/observation durations of included studies, we expressed results in terms of monthly occurrence of RTIs as well.

Our risk of bias assessment largely referred to the work by David et al. that used the Cochrane collaboration’s risk of bias tool [44,48]. Additional literature not included by their work was assessed independently. We also applied the Jadad et al. scoring method to estimate the studies’ methodological qualities [45]. Risk of publication bias across selected studies was scrutinized using funnel plots in order to detect asymmetries within trials referred to in the meta-analysis.

We quantitatively estimated effect sizes using meta-analysis and forest plots displaying odds rations (OR) and risk ratios (RR) with their respective 95% confidence intervals (CI) for binary data. For continuous parameters (e.g., duration of antibiotic therapy) we synthesized the incidence risk ratio (IRR) between groups. 

Where quantitative data was available, we synthesized the results of the included studies by meta-analysis with the R language for statistical programming version R-4.3.1 using the “meta” package. Due to heterogeneity between studies, we conservatively applied a random effect model but compared results to the fixed effect model as well. For the binary outcomes we used the “metabin” function, which uses the Mantel–Haenszel method for pooling and the DerSimonian–Laird estimator for tau². For measures of event counts, the “metafor” function was used. Between study heterogeneity was assessed using the I² statistic [57,58].

Analogous to David et al. [44], we deduced the number of participants with ≥1 infection from the total number of infections occurring and the number of subjects with recurring infections/relapses. Information pertaining to the occurrence of episodes was principally retrieved from David et al. after confirmation regarding where data was available. In contrast to a Cochrane review by Karsch–Voelk et al. [52], we included Spasov et al. [41] in our analysis, who compared to standard treatment instead of placebo, as well as trials published thereafter. Melchart, Bräuning, Turner (2005), Sumer and Forth et al. used multiple Echinacea species, extraction methods or dosage strengths [15,17,33,36,37]. We pooled effects from the treatment groups into one comparison each. Sumer et al. [37] used 4 arms comparing increased dosing during acute RTI episodes with a low, preventative dosage [36]. The latter was conservatively considered as the control treatment. Vonau and Coegniet et al. [59,60] studied preventive applications of *Echinacea* for urinary tract infections and were therefore excluded, as were studies investigating anything other than RTIs. Cohen et al. [18] reported a number of subjects experiencing otitis media, tonsillopharyngitis or pneumonia individually, and we calculated the mean of subjects experiencing any of the three complications.

This work intentionally aimed to survey a wide range of studies in the primary analysis to obtain a general overview on preparations containing *Echinacea* at first. Consequently, we included non-treatment controlled or actively controlled studies only if appropriately randomized. We did not restrict the study to a single *Echinacea* species or manufacturing technique, and preparations that contained further ingredients like zinc, other herbs or vitamins were included. We collected information on RTIs, recurrent RTIs/relapses, RTI complications and antibiotic therapies reported from the time of the onset of *Echinacea* intake until the end of follow up during the studies. Our investigation was in alignment with the latest recommendations by the PRISMA working group for reporting meta-analyses [61].

Finally, we decided ad hoc to investigate the safety profile of *Echinacea* while comparing the occurrence of adverse events during intervention. For this parameter, we solely referred to comparisons of *Echinacea* versus placebo in healthy subjects. Trials in patients with underlying illness like cancer, with concomitant antibiotic therapy or comparisons to oseltamivir, were excluded as they were expected to skew the basis for establishing the net effect of *Echinacea* with respect to safety. 

## Figures and Tables

**Table 1:** Example search strategy.

Step	Search
1	Echinacea.mp. or exp Echinacea/
2	coneflower.mp.
3	Black Sampson.mp.
4	1 or 2 or 3
5	(randomized controlled trial or controlled clinical trial).pt. or randomized.ab. or randomized.ab. or placebo.ab. or drug therapy.fs. or randomly.ab. or trial.ab. or groups.ab.) not (exp animals/ not humans.sh.)
6	4 and 5
7	Remove duplicates from 6
8	limit to controlled, randomized human RTI studies

**Figure A2:**
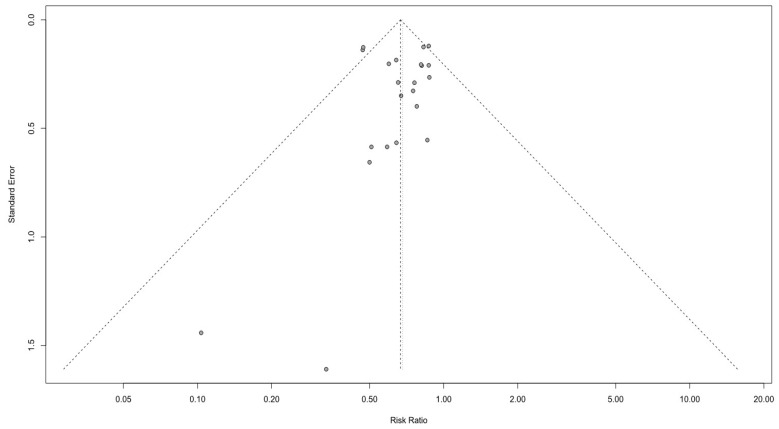
Funnel plot in detail referred to Figure 2 of main text.

**Figure A3:**
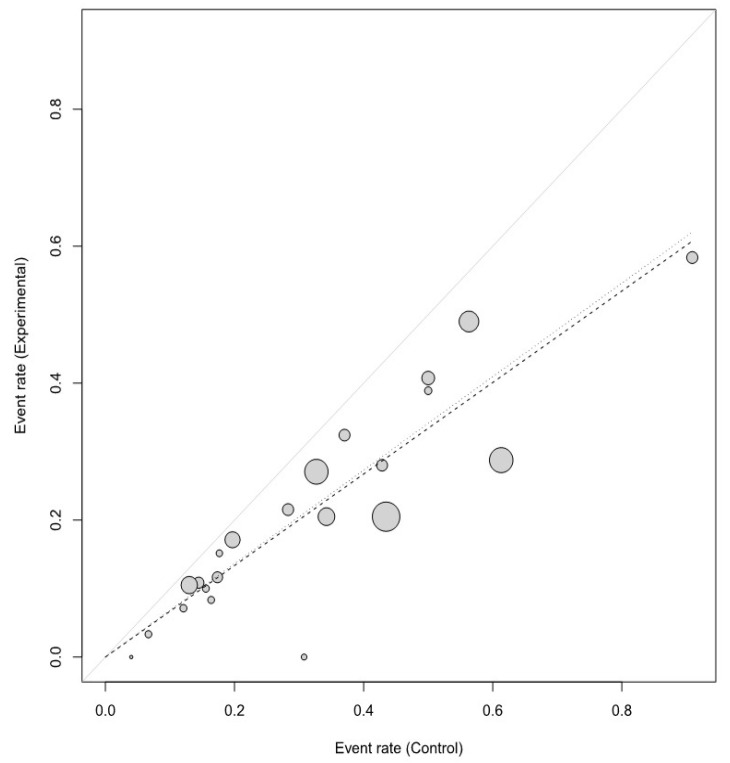
Abbey plot in detail referred to Figure 2 of main text.

**Figure A4:**
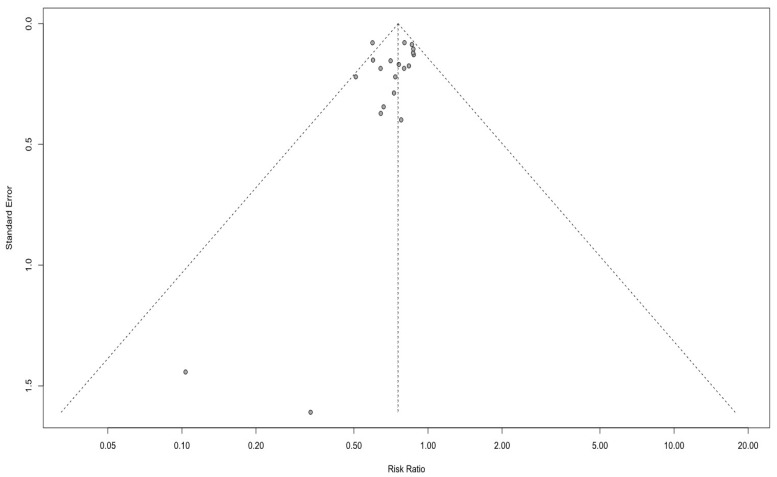
Funnel plot in detail referred to Figure 3 of main text.

**Figure A5:**
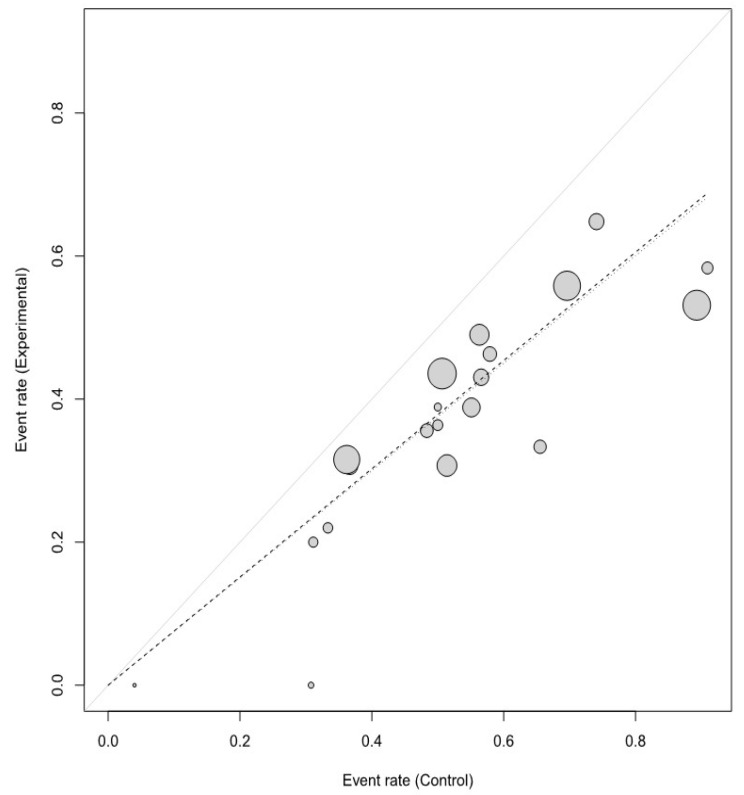
Abbey plot in detail referred to Figure 3 of main text.

## Data Availability

All articles reviewed have been included in the reference list.

## Appendix A

**Table A1:** Description of included studies and assessment of methodological quality according to Jadad scoring [45].

Study/ Registry	Echinacea Species	Control	Extraction Method	Supplement	Duration of Treatment/Observation	Daily Dose/Amount of Echinacea [mg]	Participant Number (N, ITT)	Age [Years]	Cold Definition	Jadad Score [18]
Bendel R et al., 1988 [20]	EPAr + EPUr Esberitox	NT	Ethanolic Extract	Thuiae occid, Baptisia	50 days in addition to Chemotherapy Prevention	3 × 50 drops	50	>18	Respiratory Infection induced Stop of Chemotherapy medically confirmed	2
Bendel R et al., 1989 [19]	EPAr + EPUr Esberitox	NT	Ethanolic Extract	Thuiae occid, Baptisia	12 Chemotherapy Cycles à 14 days Prevention	3 × 25 drops	67	>18	Respiratory Infection induced Stop of Chemotherapy medically confirmed	1
Bräuning B et al., 1992 [36]	EPUr	Placebo	Ethanolic Extract	None	Therapy 8–10 days	Dosis 1 = 90 drops/450 mg Dosis 2 = 2 × 90 drops/900 mg	180	18–60	Flu-like Infections, clinically confirmed (virally vs. bacterial)	1
Cohen HA et al., 2004 [18]	EPU + EAN	Placebo	Glycerol extract	Propolis + Vitamin C	3 mts Prevention	2–4 × 5–7.5 mL 500–1500 mg	328	1–5	Patient reported- medically confirmed	4
Forth H, Beuscher N, 1981 [17]	EPAr + EPUr (Esberitox)	Placebo (20 mg Vit C)	Ethanolic extract	Thuiae occid, Baptisia	3 × 14d cycles for up to 17 weeks Prevention	3 × 25 drops or 3 × 1 tablet	95	>18	Patient reported Rhinitis	1
Freyer HU, 1974 [22]	EPAr + EPUr	NT	Ethanolic extract	Thujae occid, Baptisia	6 weeks Prevention	3 × 20 drops	284	6–17	“infections” not further described	1
Goel V et al., 2004 [38]	EPU	Placebo	Ethanol	None	7 days Therapy	1st day: 10 × 4 mL 6 days: 4 × 4 mL	128 ≥2 colds/y	18–65	Patient reported Confirmed by study nurse/physician	5
Grimm (1999)/Schoeneberger (1996) [23,62]	EPUh	Placebo	Pressed-juice	None	2 mts Prevention	2 × 4 mL 6200 mg ^2)^	108 ≥3 colds/y	>11	Patient reported- Confirmed by physician	5
Hall, H et al., 2007 [24]	EPUh	Placebo	Pressed Juice	none	28 days Prevention	4 × 2 capsules/8000 mg	32	>17	Incidence of URTI Patient-reported outcome	4
Helbig (1961) [25]	EPUr + EANr (Esberitox)	NT	Ethanolic extract	Thujae occid/Baptisia	1 mt Prevention	3 × 20 drops	644	1–3	Infections of Upper Respiratory Tract	0
Jawad et al., 2012 [26]	EPU h + r (Echinaforce)	Placebo	Ethanolic extract	None	4 mts Prevention	3–5 × 0.9 mL 2.7–4.5 mL 2400–4000 mg	717	>17	Patient reported–confirmed by Jackson method Virally confirmed infections	5
König D, 1999 or Berg A (1998) [21]	EPUh	Placebo/Magnesium	Pressed Juice i.c. placebo and Biomagnesin	None	28 days Prevention	3 × 40 drops/8000 mg	42 (Athletes)	>17	Incidence of URTI Infection, Training failures	3
Kolev E et al., 2022 [27]	EPU h + r (Echinaforce)	NT	Ethanolic extract	None	5 months Prevention	3–5 × 2 tablets (400 mg)/2400–4000 mg	119	18–75	Patient reported, physician and virally-confirmed infections	2
Melchart (1998) 3-arm study [15]	EPUr	Placebo	Ethanolic extract	None	3 mts Prevention	2 × 50 drops 1800 mg ^3)^	99 (90 placebo) =/>3 colds/y	18–65	Patient reported- Confirmed by physician	4
Melchart (1998) 3-arm study [15]	EANr		Ethanolic extract	None	3 mts Prevention	2 × 50 drops 1800 mg ^3)^	100 (90 placebo)	18–65	Patient reported- Confirmed by physician	
O’Neil J et al., 2008 [28]	EPU	Placebo	Dried Echinacea, not specified	None	8 weeks Prevention	3 × 2 capsules/1800 mg	90	18–65	Patient reported- Study staff confirmed	4
Ogal M et al., 2021 NCT02971384 [14]	EPU h + r (Echinaforce)	Placebo (VitC)	Ethanolic extract	None	4 months Prevention	3–5 × 1 tablet (400 mg)/1200–2000 mg	203	4–12	Patient reported, physician and virally-confirmed infections	5
Awad OG, 2020 2015NBA5732814 [29]	EPU root	Azithromycin (AZT) vs. NTC	Powder	None	6 × 10 days over 6 months Prevention	3 × 5 mL (250 mg)/1500 mg + AZT I.c. no prevention/ATZ prevention	300	5–16	Recurrent tonsillitis, reported by parents	1
Schmidt U et al., 1990 [30]	EAN	Placebo	Ethanolic extract	Eupatorium/Baptisia	2 month Prevention	1 × 12 mL/1440 mg ^1)^	609	>17	Patient reported- Confirmed by physician	4
Schulten B et al., 2001 [40]	EPUh (Echinacin^®^)	Placebo	Pressed Juice	None	10 days Therapy	2 × 5 mL (7750 mg)	80	>17	Patient reported confirmed by Jackson method (full picture of cold)	3
Spasov AA et al., 2004 [41]	EPUh	NT (standard therapy)	Pressed Juice	None (i.a. std treatment)	10 days Therapy	3 × 10 drops	80	4–11	Patient reported, Physician confirmed uncomplicated RTIs	2
Sperber SJ et al., 2004 [31]	EPUh Echinaguard	Placebo	Pressed juice	None	14d Prevention	3 × 2.5 mL	46	18–65	Artificially Rhinovirus Infection, Jackson definition	3
Sumer J et al., 2023 [37]	EPU (h + r) (Echinaforce)		Ethanolic extract	None	10 days Therapy	1–5 tablets (3360 mg) or 2–7 sprays (1120 mg) 3360–16,800 mg	246	>17	Patient reported, physician and virally-confirmed flu-like infections	4
Tiralongo E et al., 2012 [32]	EPUr + EANr (MediHerb)	Placebo	Ethanolic extract	None	5–9 weeks Prevention	Priming dose 2 × 1 tabs followed by exposition dose 2 × 2 tabs sick dose 3 × 2 tabs/3825 mg and 7650 mg	148	18–65	Natural exposition (air travel)	5
Turner RB et al., 2005 [16] 4-arm study	EANr	Placebo	20% Ethanolic extract	None	7 days Prevention 5 days Therapy	3 × 1.5 mL (300 mg)/900 mg	206	>17	Artificially Rhinovirus Infection, Jackson definition Patient reported, physician and virally-confirmed flu-like infections	4
Turner RB et al., 2005 [16] 4-arm study	EANr	Placebo	60% Ethanolic extract	None	7 days Prevention 5 days Therapy	3 × 1.5 mL (300 mg)/900 mg	203	>17		4
Turner RB et al., 2005 [16] 4-arm study	EANr	Placebo	CO_2_ extract	None	7 days Prevention 5 days Therapy	3 × 1.5 mL (300 mg)/900 mg	196	>17		4
Turner RB et al., 2000 [33]	EPU	Placebo	Powder Almost no alkylamides	None	19 days (14 days prevention + 5 days therapy) Prevention	3 × 1 capsule/900 mg	92	>17	Artificial Rhinovirus Infection,	3
Taylor (2003)/Weber (2005) [35,42]	EPUh (Echinacin^®^)	Placebo	Pressed juice	None-	4/1 week Therapy	2 × 3.75–5 mL 7.5–10 mL 7500–10,000 mg	407/401	2–11	Study staff confirmed	5
Yakoot M et al., 2011 [43]	E (Immumax)	Placebo	Extract	Garlic, Nigella sativa, Panax ginseng, Vitamin C, Zinc	14 days Therapy	2 × 1 capsule (120 mg)/240 mg	63	38 (Mean)	Patient reported- Confirmed by physician	5
Zhang X et al., 2003 [34]	EPUr	Placebo	Powdered root	None	8 weeks Prevention	2 × 1 capsule (294 mg)/588 mg	111	18–65	Patient reported- Confirmed by physician	3

E…Echinacea (not further specified) EAN…*Echinacea angustifolia*. EPU…*Echinacea purpurea*. h…herb. r…root. NT…No-treatment control. ITT…Intention-to-treat. ^1)^ with 120 mg/mL EA extract; ^2)^ product contains 22% Ethanol for stabilisation; ^3)^ at 20 drops / ml and δ = 0.9 gr/mL.

**Table A2:** Methodological Quality Assessment according to Cochrane Risk of Bias Tool and Jadad Scoring [46,55].

Study	Random Sequence Generation	Allocation Concealment	Blinding Patients/Personel	Blinding Outcome Assess	Incomplete Outcome	Select Reporting	Other Bias	Overall Jadad [0–5]		
Bendel, 1988 [20]	+	+	-	-	+	+	+	2	-	=High Risk
Bendel, 1989 [19]	?	+	-	-	+	+	+	1		
Bräunig, 1992 [36]	?	?	?	?	+	-	+	1	+	=Low Risk
Cohen, 2004 [18]	+	+	+	+	?	+	+	4		
Forth, 1981 [17]	+	-	-	?	?	-	?	1	?	=Unclear
Freyer, 1974 [22]	+	-	-	-	+	+	+	1		
Goel, 2004 [38]	+	+	+	+	+	+	+	5		
Grimm, 1996 [23]	+	?	+	+	+	+	+	5		
Hall, 2007 [24]	+	+	+	+	?	?	+	4		
Helbig, 1961 [25]	?	-	-	-	+	+	+	1		
Jawad, 2012 [26]	+	+	+	+	?	?	+	5		
Kolev, 2022 [27]	+	-	-	+	+	+	+	2		
Berg, 1998 [21]	?	+	+	?	+	+	+	3		
Melchart, 1998 [15]	+	+	-	?	+	+	+	4		
Ogal, 2021 [14]	+	+	?	+	+	+	+	5		
O’Neil, 2008 [28]	+	+	+	+	-	+	+	4		
Osama 2020 [29]	+	+	-	-	-	+	?	1		
Raus, 2015 [39]	+	+	+	+	+	+	+	5		
Schmidt 1990 [30]	?	+	?	?	+	+	+	4		
Schulten, 2001 [40]	+	-	+	+	+	?	+	3		
Spasov, 2004 [41]	+	-	-	-	?	?	?	2		
Sperber, 2004 [31]	?	?	+	+	+	+	+	3		
Sumer, 2023 [37]	+	+	?	?	+	+	+	4		
Taylor03-Weber05 [35,42]	+	+	+	+	+	+	+	5		
Tiralongo, 2012 [32]	+	+	+	+	?	+	+	5		
Turner, 2000 [33]	?	?	?	+	+	+	+	3		
Turner, 2005 [16]	+	?	+	+	+	+	+	4		
Yakoot, 2011 [43]	+	+	+	+	+	?	+	5		
Zhang, 2003 [34]	+	+	-	-	?	+	+	3		

**Table A3:** Results overall.

	RTIs/Pts with RTIs		Recurrent RTIs/Pts with Recurrent RTIs		Complications/Pts with Complications		Pts with AB/AB Treatment Days/Mean Difference [Days]		Adverse Events (Number of Events)	
Study Registry	Echinacea (N)	Control (N)	Echinacea	Control	Echinacea	Control	Echinacea	Control	Echinacea	Control
Bendel R et al., 1988 [20]	24/12 (33)	30/17 (34)	-	-	-	-	-	-	Safety of Echinacea during Chemotherapy not assessed	
Bendel R et al., 1989 [19]	0/0 (25)	1/1 (25)	-	-	-	-	-	-	Safety of Echinacea during Chemotherapy not assessed	
Bräunig B et al., 1992 [36]	-	-	-	-	1/2 (120)	4/3 (60)	0/0	1/-	4	5
Cohen HA et al., 2004 [18]	138/85 (160)	308/150 (168)	53	158	54/18 (160)	136/45 (168)	-/541/3.40 (160)	-/1084/6.50 (168)	9	7
Forth H, Beuscher N, 1981 [17]	22/22 (66)	19/19 (29)							None reported	None reported
Freyer HU, 1974 [22]	43/43 (140)	74/74 (144)							0	0
Goel V et al., 2004 [38]	-	-	-	-	2/2 (59)	5/5 (69)	-	-	8	6
Grimm (1999)/Schoeneberger (1996) [23,62]	35/42 (54)	40/50 (54)	14/7 (54)	18/8 (54)	37 (54)	54 (54)	-	-	11	7
Hall, H et al., 2007 [24]	7/7 (18)	7/7 (14)	-	-	-	-	-	-	Not reported	Not reported
Helbig 1961 [25]	66/- (322)	140/- (322)	66	140	-	-	15 (322)	41 (322)	0	0
Jawad (2012) [26]	149/112 (355)	188/131 (362)	65/28 (355)	100/43 (362)	5/5 (355)	7/7 (362)	1/7/0.02 (355)	4/33/0.09 (362)	293	306
König D, 1999 or Berg A (1998) [21]	0/0 (14)	4/4 (13)	-	-	0/0 (14)	1/1 (13)	-	-	0	3
Kolev E et al., 2022 [27]	21/21 (59)	29/29 (60)	-	-	0/0 (59)	2/2 (60)	8 (59)	12 (60)	3	5
Melchart (1998) 3-arm study [15]	-	-	4/4 (EPUr) (99)	6/6 (90)	-	-	-	-	13	12
Melchart (1998) 3-arm study [15]	-	-	7/7 (EAN) (100)		-	^-^	-	-	21	
O’Neil J et al., 2008 [28]	9/9 (45)	14/14 (45)	-	-	-	^-^	-	-	(8%) 2	(7%) 2
Ogal M et al., 2021 NCT02971384 [14]	61/40 (103)	86/54 (98)	21/16 (103)	32/22 (98)	11/10 (103)	30/20 (98)	6/45/0.44 (103)	15/216/2.20 (98)	76	105
Awad OG, 2020 2015NBA5732814 [29]	2/- (100)	4/- (100)	-	-	2 (100)	4 (100)	-	-	Not assessed as in combination with AZT	
Raus K et al., 2015 EUDRA-CT 2010-021571-88 [39]	-	-	-	-	5/5 (203)	9/9 (217)	4 (203)	4 (217)	Not assessed as in comparison with Oseltamivir	
Schmidt U et al., 1990 [30]	164/132 (303)	199/155 (306)	32	44					12	10
Schulten B et al., 2001 [40]	-	-			1/1 (41)	4/4(39)			8	9
Spasov AA et al., 2004 [41]	-	-					1 (41)	1 (39)	1	0
Sperber SJ et al., 2004 [31]	14/14 (24)	20/20 (22)							2	4
Sumer J et al., 2023 [37]	-	-			0/0 (61)	2/2 (64)	0/0/0 (61)	2/6/0.09 (64)	Comparison of different Echinacea galenic forms, no non-Echinacea reference.	
Tiralongo E et al., 2012 PHM0608HREC [32]	31/31 (72)	43//43 (76)							3	2
Turner RB et al., 2000 [33]	11/11 (50)	14/14 (42)							0 No significant side effect seen	0
Turner RB et al., 2005 [16]	73/73 (149)	58/58 (103)							2% (prevention phase)	2% (prevention phase)
Taylor (2003) [42] Weber (2005) [35]	- -	- -	137/110 (200)	163/142 (207)			30 (200)	27 (207)	152	146
Yakoot M et al., 2011 [43]	-	-			0/0 (31)	2/2 (32)	0 (31)	2 (31)	No significant difference between groups but no listing of AEs	
Zhang X et al., 2003 [34]	25/44 (54)	33/57 (57)							15	4

Pts…Participants. RTI…Respiratory Tract Infections. AB…Antibiotics.

**Table A4:** Results from sub-analysis with resulting risk ratios per analysis section.

Subanalysis	Subjects with RTI	Monthly Risk of RTI	Overall Infections	Subjects with Recurrent RTI	Number of Recurrent RTI	Subjects with Complication	Number of Complications	Subjects with AB	Overall AB Days
Overall Result	0.75 [0.69–0.81] *I*^2^ = 27%	0.68 [0.61–0.77] *I*^2^ = 29%	0.75 [0.69–0.82] *I*^2^ = 55%	0.77 [0.68–0.88] *I*^2^ = 0%	0.60 [0.46–0.80] *I*^2^ = 88%	0.44 [0.36–0.54] *I*^2^ = 0%	0.52 [0.43–0.64] *I*^2^ = 32%	0.60 [0.39–0.93] *I*^2^ = 34%	0.30 [0.12–0.73] *I*^2^ = 91%
Jadad Score ≥ 4	0.78 [0.71–0.86] *I*^2^ = 40%	0.75 [0.64–0.87] *I*^2^ = 32%	0.84 [0.80–0.88] *I*^2^ = 0%	0.77 [0.68–0.88] *I*^2^ = 0%	0.63 [0.46–0.87] *I*^2^ = 88%	0.47 [0.37–0.58] *I*^2^ = 0%	0.53 [0.41–0.68] *I*^2^ = 52%	0.77 [0.34–1.45] *I*^2^ = 34%	0.30 [0.12–0.73] *I*^2^ = 91%
Lipophilic Extracts	0.75 [0.66–0.83] *I*^2^ = 47%	0.66 [0.56–0.78] *I*^2^ = 50%	0.72 [0.64–0.81] *I*^2^ = 68%	0.63 [0.51–0.78] *I*^2^ = 0%	0.53 [0.39–0.73] *I*^2^ = 72%	0.46 [0.36–0.58] *I*^2^ = 0%	0.42 [0.35–0.50] *I*^2^ = 0%	0.45 [0.30–0.66] *I*^2^ = 0%	0.30 [0.12–0.73] *I*^2^ = 91%
Hydrophilic Extracts	0.79 [0.67–0.94] *I*^2^ = 0%	0.75 [0.56–1.02] ns, 0%	0.82 [0.66–1.02] ns, *I*^2^ = 15%	0.80 [0.67–0.96] *I*^2^ = 0%	0.87 [0.66–1.14] Ns, *I*^2^ = 0%	0.26 [0.05–1.26] Ns, *I*^2^ = 0%	0.68 [0.50–0.92] *I*^2^ = 0%	1.14 [0.76–1.73] *I*^2^ = 0%	No study providing data
Echinacea only	0.80 [0.75–0.85] *I*^2^ = 0%	0.78 [0.71–0.85] *I*^2^ = 0%	0.79 [0.74–0.85] *I*^2^ = 7%	0.77 [0.68–0.88] *I*^2^ = 0%	0.80 [0.67–0.96] *I*^2^ = 16%	0.47 [0.34–0.65] *I*^2^ = 0%	0.64 [0.54–0.77] *I*^2^ = 0%	0.73 [0.41–1.33] Ns, *I*^2^ = 26%	0.21 [0.15–0.29] *I*^2^ = 0%

Green 0–40% low heterogeneity. Rose 41–60% moderate heterogeneity. Light Red 61–75% substantial heterogeneity. Dark Red > 75% Considerable heterogeneity or non-significant result.
